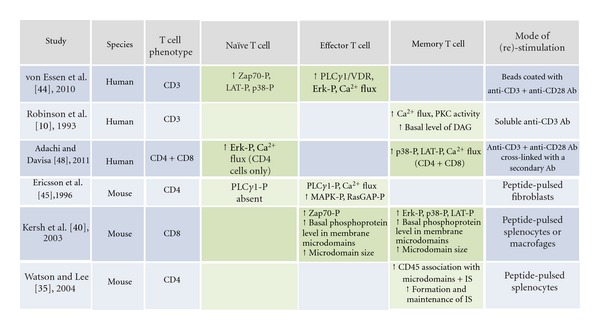# Mechanisms behind Functional Avidity Maturation in T Cells

**DOI:** 10.1155/2012/163453

**Published:** 2012-04-26

**Authors:** Marina Rode von Essen, Martin Kongsbak, Carsten Geisler

**Affiliations:** Department of International Health, Immunology and Microbiology, Faculty of Health Sciences, University of Copenhagen, Blegdamsvej 3, DK-2200 Copenhagen, Denmark

## Abstract

During an immune response antigen-primed B-cells increase their antigen responsiveness by affinity maturation mediated by somatic hypermutation of the genes encoding the antigen-specific B-cell receptor (BCR) and by selection of higher-affinity B cell clones. Unlike the BCR, the T-cell receptor (TCR) cannot undergo affinity maturation. Nevertheless, antigen-primed T cells significantly increase their antigen responsiveness compared to antigen-inexperienced (naïve) T cells in a process called functional avidity maturation. This paper covers studies that describe differences in T-cell antigen responsiveness during T-cell differentiation along with examples of the mechanisms behind functional avidity maturation in T cells.

## 1. Introduction

T lymphocytes are very potent cells that play key roles in our immune system; without T cells we would quickly die from infection. The T cells patrol our organism to guard us against pathogenic microorganisms as part of adaptive immunity. In secondary lymphoid organs, such as lymph nodes and the spleen, small peptide fragments (antigens) of the pathogens are presented to antigen-inexperienced (naïve) T cells by professional antigen presenting cells (APC). This encounter induces proliferation and differentiation of the naive T-cell into an armed T-cell population that migrates to the site of infection. Here, reencounter with the same pathogen rapidly triggers the effector function of the armed T cells resulting in elimination of the pathogen. Following antigen clearance, most of the effector T cells die leaving only a small population of memory T cells. In case of reinfection with the same pathogen, memory T cells will mount a prompt response by immediately producing effector cytokines and by rapidly proliferating into a large number of secondary effectors [1–4]. This substantial increase in antigen-responsiveness of both effector and memory T cells upon reencounter with the pathogen is a fundamental property of adaptive immunity.

## 2. The Concept of Functional Avidity Maturation

Lymphocytes recognize antigens through specialized antigen receptors. These include the B-cell receptor (BCR) on B cells and the T-cell receptors (TCR) on T cells. During the cause of an immune response, a high number of point mutations take place in the BCR genes of the dividing B cells. This result in a panel of B cells expressing BCR with varying affinities against the antigen, and the B cells carrying BCR with the highest affinity are selectively expanded. As a consequence, high-efficiency B cells are selected during the immune response in a process known as affinity maturation [5]. Unlike B cells, T cells lack the capacity to mutate their TCR genes after T-cell activation, and thus classical affinity maturation does not take place in T cells. Still, T-cell sensitivity to antigens can be extensively enhanced in antigen-experienced (primed) T cells compared to naïve T cells in a process called “functional avidity maturation” [6–13].

## 3. T-Cell Activation Signals: The Basis of Functional Avidity Maturation

### 3.1. Early Studies That Indicated the Existence of Functional Avidity Maturation

The observation that fundamental differences exist in antigen sensitivity between naïve and primed T cells was first described in the late 80′s by Cooper and coworkers. They found that only primed T cells produced IL-2 and proliferated* in vitro* in response to TCR triggering induced by anti-CD3 antibodies and monocytes [14]. Similar observations were later reported by others [7, 9–13, 15]. Cooper and co-workers also introduced the idea that signals in addition to TCR signals, here exemplified by IL-2 receptor signals, were required for activation of naïve T cells [14]. Along this line, Mark Davis' group demonstrated that in addition to TCR signals naïve T cells require costimulatory signals through CD28 to become fully activated [16]. This finding was supported in a subsequent study, where Croft et al. showed that activation of both effector and memory T cells were considerably less dependent on co-stimulatory signals than naïve T cells [9]. Several *in vivo* and *ex vivo* studies have confirmed the early observations that effector and memory T cells have a lower threshold of activation and respond more robustly than naïve T cells [12, 13, 17]. As an example, Slifka and Whitton demonstrated a 50 fold increase in T-cell responsiveness to antigen during a LCMV infection. Furthermore, they found that coengagement of the coreceptor CD8 with the TCR was required for naïve T-cell activation, whereas activation of effector T cells was relatively CD8-independent [17]. In an equivalent study also examining T-cell responses to infection, Pihlgren et al. demonstrated a similar 50-fold increase in antigen responsiveness of both effector and memory cell populations as compared to naïve cells [12]. Interestingly, a study by Mescher and co-workers suggested that memory T cells were intrinsically more sensitive to TCR stimulation than their naïve counterparts [13], adding TCR signaling to the growing list of differences between naïve and primed T cells. An overview of studies indicating the existence of functional avidity maturation is given in Table 1.

Today, it is widely accepted that T-cell activation should not be considered as a single signal process, but as a sum of interdependent signals. The current model for T-cell activation, referred to as the 3-signal model, predicts that in addition to antigen-induced TCR-triggering optimal activation of naïve T cells requires at least two additional signals. These signals are delivered through co-stimulatory receptors predominantly CD28 [18, 19] and receptors for cytokines like IL-2, IL-12, IFN-*α*, and IL-1 [20–25].

### 3.2. TCR Signal Initiation in Naïve versus Primed T Cells: The Immunological Synapse and CD28

TCR signaling takes place at the interface between the T-cell and the antigen presenting cell. At this contact zone, often referred to as the immunological synapse (IS), TCR-signaling components including the TCR itself as well as intracellular-signaling molecules are continuously accumulated during antigen contact [26]. Although somewhat controversial [26, 27], formation of an IS correlates with generation of a robust immune response, and is considered a prerequisite for T-cell activation [28, 29]. Even so, new insight into the biology of immunological synapses has revealed that TCR signaling is already initiated in TCR microclusters prior to IS formation. In a ligand-dependent manner, CD28 localizes to preformed TCR microclusters counting 11–17 TCRs [30] together with key signaling molecules [31]. Formation of the mature IS includes accumulation of hundreds of such TCR microclusters [31]. At the IS, CD28 signaling both induces structural stabilization and enlargement of the area itself [32, 33]. Formation of the IS is a mechanism shared by naïve and primed T cells; however, a mature IS is formed more quickly in primed T cells and only naïve T cells require CD28 co-stimulatory signals to form the IS [34, 35]. These observations are consistent with reports indicating that primed T cells are less dependent on CD28-costimulation than naïve T cells [9, 36–38]. Eventhough the exact implication of CD28 signaling in T-cell activation is still elusive, it is generally agreed that CD28 amplifies intracellular signaling induced by antigen-triggering of the TCR through modulation of morphological features and TCR signals [32, 33]. In addition to CD28, signaling other differences between naïve and primed T cells exists at the IS. A study by Watson and Lee illustrated that the phosphatase CD45 is a more integral component of the IS in primed T cells as compared to naïve cells [35]. CD45 is a transmembrane tyrosine phosphatase that maintains Lck activity by promoting dephosphorylation of an inhibitory carboxy-terminal tyrosine residue of Lck. Lck activity is a necessity for initiation of TCR signal transduction [39]. Interestingly, Watson and Lee also showed that CD45 is already associated with TCR microdomains in the plasma membrane prior to synapse formation in resting memory T cells in contrast to their naïve counterparts [35]. This finding parallels the study of Kersh et al. who showed that a higher basal level of phosphorylation (activation) was seen in membrane associated signaling molecules in resting primed T cells [40]. It, therefore, appears that primed T cells are in a higher “state of alert” prior to antigen encounter, correlating with the higher sensitivity of primed T cells to antigen stimulation.

### 3.3. TCR Signaling in Naïve versus Primed T Cells

In addition to differences in the organization of signaling molecules, the actual TCR signaling events induced in naïve and primed T cells following TCR triggering differs. The current model for TCR signaling postulates that following TCR triggering the tyrosine kinase Lck is activated resulting in phosphorylation of the CD3 and zeta chains of the TCR in addition to activation of Zap70 [41, 42]. Activated Zap70 phosphorylates LAT that subsequently recruits and activates several proteins including PLC-*γ*1. Activation of PLC-*γ*1 results in the hydrolysis of phosphatidylinositol 4,5-biphosphate (PIP2) to inositol 3,4,5-triphosphate (IP3) and diacylglycerol (DAG). IP3 regulates intracellular calcium mobilization, and DAG regulates the activation of PKC and contributes to Ras and mitogen-activated protein kinase (MAPK) cascade activation [41, 42]. The vast majority of studies contributing to the current model for TCR signaling were performed using immortal T-cell lines or primed T cells propagated *in vitro*. However, as significant differences in gene and protein expression exist between naïve and primed T cells [43], significant differences in TCR signaling in primed and naïve T cells could be imagined. By studying naïve human T cells isolated from freshly drawn blood samples, we have recently shown that the classical model for TCR signaling must be revised as naïve T cells only express PLC-*γ*1 at very low levels compared to primed (effector) T cells. Following *in vitro* priming, PLC-*γ*1 was upregulated approximately 75 fold, an upregulation that correlated with greater TCR responsiveness [44]. One of the striking signaling differences that we and others have observed between naïve and primed T cells is a strongly diminished ability of naïve T cells to flux calcium in response to TCR triggering [10, 44, 45]. The very low expression of PLC-*γ*1 in naïve T cells could explain the impaired calcium flux in these cells [44]. Based on previous studies demonstrating that vitamin D can up-regulate PLC-*γ*1 in other cell types [46, 47], we investigated if vitamin D via the vitamin D receptor (VDR) was responsible for PLC-*γ*1 up-regulation during T-cell priming. Indeed, we found that VDR was quickly up-regulated following TCR triggering and that induction of VDR was required for PLC-*γ*1 up-regulation. As PLC-*γ*1 is a central molecule in the classical TCR signaling pathway and is weakly expressed in naïve human T cells, we wondered which signaling events could be responsible for the activation-induced VDR up-regulation. We found that the nonclassical TCR signaling pathway in which Zap70 directly activates p38-induced VDR expression. We further found that whereas activation of Zap70 and p38 was at least as efficient in naïve T cells as in primed T cells following TCR triggering, activation of Erk was significantly reduced in naïve T cells. Thus, our study demonstrated that fundamental differences exist in the signaling pathways between naïve and primed T cells. 

Adachi and Davis also compared TCR signaling in human naïve and primed (memory) T cells. In contrast to us, they found a stronger Erk activation along with lower activation of Zap70 and p38 in naïve T cells as compared to primed cells. They proposed that the strong Erk activation observed in naïve T cells disrupted early TCR signaling events as part of a negative feedback mechanism [48]. The discrepancy between the two human studies might be due to two different primed T-cell populations studied (effector and memory cells, resp.); however, it might also be explained by the different modes of TCR triggering used. In our study, purified naïve human T cells were stimulated using beads coated with anti-CD3 and anti-CD28 antibodies. Adachi and Davis used high concentrations of soluble anti-CD3 and anti-CD28 antibodies cross-linked by secondary antibodies to stimulate the T cells. By using cross-linked antibodies for stimulation, a very strong receptor signaling is achieved. As illustrated in a series of mouse virus studies, the strength of TCR signaling determines the requirement for additional activation signals like CD28 signaling and also results in somewhat different responses [19]. In line with this, Adachi and Davis found that naïve CD4 T cells could flux calcium when their stimulation protocol was used, implying very strong signaling and the need for a fast negative feedback mechanism. Both scenarios could be relevant for human immunity where a wide range of pathogens with different origins is encountered.

A few studies investigating TCR signaling events in naïve versus primed T cells have also been conducted in mice [40]. Unfortunately, mouse and man seem to differ when it comes to some of the signaling molecules involved in TCR signaling. In contrast to human T cells, naïve and primed mouse T cells seem to express similar levels of both VDR and PLC-*γ*1 [45, 49]. Even so, studies on mice T cells have found that it is only in primed T cells that TCR triggering induces phosphorylation of PLC-*γ*1 and subsequent calcium flux [45] as found for human T cells. It is, therefore, likely that despite a different “route of action” the outcome are the same concerning the ability to flux calcium in T cells from man and mice.

Collectively, these studies illustrate fundamental differences in TCR signaling pathways between naïve and primed T cells, differences based in particular on the lack of naïve T-cell signaling molecules used by the primed T cells. A detailed overview of the published differences in the signaling machinery in naïve versus effector and memory T cells is given in Table 2.

### 3.4. Cytokines as the “Third” Activation Signal in Naïve versus Primed T Cells

Within the last years, the importance of cytokine receptor signaling as a “third-signal” in activation of naïve T cells has been acknowledged. The requirement for a “signal 3” mediated by inflammatory cytokines is considered a mean for T cells to determine if “danger” is present [50]. Although both naïve CD4 and CD8 T cells are dependent on these “danger signals” for full activation, they differ in their requirement for specific cytokines. Early studies describing a need for a third-signal cytokine came from a series of *in vitro *and* in vivo* experiments performed by Mesher and co-workers. They found that IL-12 and IFN-*α* provided a signal that along with antigen and CD28 signaling was crucial for naïve CD8 T-cell expansion and differentiation [51–53], findings that were validated by other groups [23, 54–57]. Eventhough IL-12 has a role in skewing the CD4 T-cell response, it has no effect on CD4 T-cell proliferation and differentiation in response to antigen. In contrast, IL-1 enhances *in vivo* expansion and differentiation of naïve CD4 T cells [58], both by acting directly on the CD4 T cells [24] and through APC modifications [25]. No studies have described a need for “the third-signal” in activation of primed T cells, but a role for IFN-*α* in homeostatic proliferation and maintenance of memory CD8 T cells has been demonstrated [59]. Thus, even though primed T cells to some extent rely on both IFN-*α* [59] and CD28 [19] for their continuous survival and antigen recognition, primed T cells clearly do not have the same prerequisite for cytokine and CD28 signaling as naïve T cells to be activated. The present literature, therefore, clearly states that the demand for the “3 signals” in T-cell activation greatly differs between naïve and primed T cells.

## 4. Molecular Mechanisms of Functional Avidity Maturation

**Figure 1:**
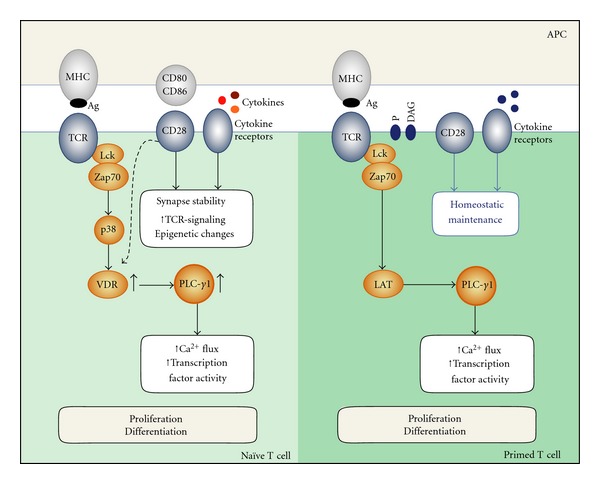
Simplified model illustrating the differences in T-cell signaling between naïve and primed T cells. In naïve human T cells, TCR engagement leads to activation of p38 through Zap70 resulting in upregulation of VDR and then PLC-*γ*1 mandatory for the naïve T cells to be activated. For activation, naïve T cells also require CD28 and cytokine receptor signals to induce and stabilize membrane structures and intracellular signaling molecules. In contrast, primed T cells already express PLC-*γ*1, have a higher DAG and phosphoprotein (P) basal level in specialized membrane structures with a high association of the CD45 molecule. In addition, signaling in primed T cells is rather independent of CD28 costimulatory signals as well as “third-signal” inflammatory cytokines, overall leading to a far more prompt antigenic response.

As discussed in this paper and summarized in Figure 1, fundamental differences in activation of naïve and primed T cells exist. This includes both the requirement for the three antigenic-induced signals as well as intrinsic differences in the signaling machinery. CD28 and cytokine receptor signaling are central components of naïve T-cell activation as they help induce and stabilize both membrane structures and intracellular signaling molecules crucial for T-cell activation. In this way, the signaling machinery is already optimized for signal transduction in primed T cells prior to antigen reencounter. As a result, primed T cells respond much faster and stronger when an antigen is eventually engaged. It therefore seems as the T cells retain a permanent imprint of a prior response to antigen. But how is such an imprint formed? Accumulating evidence suggest that epigenetic changes are likely to be a contributing factor. For example, Northrop et al. demonstrated that stable demethylation of the regulatory region of the IL-2 gene takes place during priming of naïve T cells resulting in a gain of IL-2 expression in the primed T cells [60], a discovery validated by Murayama and co-workers [61]. In addition, Thomas et al. published the observation that CD28 costimulation during T-cell priming induces a stable histone acetylation and demethylation at the IL-2 promoter, suggesting that CD28 in part function through epigenetic mechanisms [62]. A personal observation of ours shows that CD28 signaling greatly increases the TCR induced upregulation of VDR in naïve T cells. In parallel with this, Kim et al. recently published that transcription of the gene CYP27B1 is controlled by methylation of its promoter [63]. The CYP27B1 gene product controls synthesis of active vitamin D, which is a prerequisite for VDR activity and hence for upregulation of PLC-*γ*1 in naïve T cells. Moreover, it has been speculated that the “third-signal” cytokines IL-12 and IFN-*α* drive chromatin remodeling events during initial priming of naïve T cells [50]. It therefore seems likely that the more rapid and robust responses of primed T cells in comparison to naïve cells partly are a result from epigenetic changes in crucial genes, and furthermore that these changes may be driven by CD28 costimulation and “third-signal” cytokines during the initial priming phase. Despite the progress made in recent years, we still lack a clear understanding of some of the key aspects of functional avidity maturation. A better understanding of the molecular mechanisms involved in improving antigen-specific T-cell responses would be of great therapeutic value, for example, to advance vaccine efficiency.

## Figures and Tables

**Table 1:** Studies describing differences in antigen sensitivity between naïve and primed T cells. Differences listed are in comparison to naïve T cells.

Study	Species	T-cell phenotype	Effector T cell	Memory T-cell	Mode of (re)-stimulation
Slifka and Whitton [17], 2001	Mouse	CD8	>50 fold ↑ Ag responsiveness	>50 fold ↑ Ag responsiveness	Peptide antigen
Pihlgren et al. [12], 1996	Mouse	CD8	50 fold ↑ Ag responsiveness (proliferation)	50 fold ↑ Ag responsiveness (proliferation)	*In vivo* or peptide-pulsed splenocytes
Curtsinger et al. [13], 1998	Mouse	CD8		↑ Ag responsiveness (e.g., proliferation)	Beads coated with MHC/peptide
Robinson et al. [10], 1993	Human	CD3		↑ Responsiveness to TCR triggering (e.g., proliferation)	Soluble anti-CD3 Ab
Sanders et al. [7], 1989	Human	CD3		↑ Responsiveness to TCR triggering (e.g., proliferation)	Soluble anti-CD3 Ab
Schwinzer et al. [11], 1994	Human	CD3		↑ Proliferation	Anti-CD3 Ab + APC
Byrne et al. [14], 1988	Human	CD4		↑ Proliferation	Anti-CD3 Ab + APC
Croft et al. [9], 1994	Mouse	CD4	↑ Proliferation	↑ Proliferation	Anti-CD3 Ab + APC lacking co-stimulation
Luqman and Bottomly [8], 1992	Mouse	CD4		↑ Proliferation	Anti-CD3 Ab + APC lacking co-stimulation

**Table 2:** Studies describing differences in the TCR signaling machinery of naïve and primed T cells. Green cells indicate the investigated T cell populations. Arrows indicate an increase. P denotes phosphorylation of the given enzyme following TCR triggering.